# Transcription and proteome changes involved in re-innervation muscle following nerve crush in rats

**DOI:** 10.1186/s12864-022-08895-w

**Published:** 2022-09-22

**Authors:** Haotao Li, Wanqiong Yuan, Yijian Chen, Bofu Lin, Shuai Wang, Zhantao Deng, Qiujian Zheng, Qingtian Li

**Affiliations:** 1 Department of Orthopedics, Guangdong Provincial People’s Hospital, Guangdong Academy of Medical Sciences, 106, Zhongshan Road, Yuexiu District, Guangzhou, People’s Republic of China; 2 Shantou University Medical College, Shantou, People’s Republic of China; 3 Department of Orthopedics, Peking University Third Hospital, Beijing, People’s Republic of China; 4 Beijing Key Laboratory of Spinal Disease, Beijing, People’s Republic of China; 5 Engineering Research Center of Bone and Joint Precision Medicine, Beijing, People’s Republic of China; 6 Department of Orthopedics, Shantou Central Hospital, Shantou, Guangdong People’s Republic of China

**Keywords:** Re-innervation, Denervation, Peripheral nerve injury, Skeletal muscle atrophy, Transcriptomics, Proteomics

## Abstract

Severe peripheral nerve injury leads to the irreparable disruption of nerve fibers. This leads to disruption of synapses with the designated muscle, which consequently go through progressive atrophy and damage of muscle function. The molecular mechanism that underlies the re-innervation process has yet to be evaluated using proteomics or transcriptomics. In the present study, multi-dimensional data were therefore integrated with transcriptome and proteome profiles in order to investigate the mechanism of re-innervation in muscles. Two simulated nerve injury muscle models in the rat tibial nerve were compared: the nerve was either cut (denervated, DN group) or crushed but with the nerve sheath intact (re-innervated, RN group). The control group had a preserved and intact tibial nerve. At 4 weeks, the RN group showed better tibial nerve function and recovery of muscle atrophy compared to the DN group. As the high expression of *Myh3, Postn, Col6a1 and Cfi*, the RN group demonstrated superior re-innervation as well. Both differentially expressed genes (DEGs) and proteins (DEPs) were enriched in the peroxisome proliferator-activated receptors (PPARs) signaling pathway, as well as the energy metabolism. This study provides basic information regarding DEGs and DEPs during re-innervation-induced muscle atrophy. Furthermore, the crucial genes and proteins can be detected as possible treatment targets in the future.

## Background

Skeletal muscle denervation is a common disease caused by trauma, inflammation, or disease. It is well-known that denervated muscle undergoes atrophic changes, involving disruption of sarcomere and myofibrilla, and the degree of atrophy increases with the duration of denervation [1]. In order to analyze the gene expression profiles induced by denervated muscle atrophy, microarray analysis has been used in the previous survey, as well as the RNA sequencing (RNA seq) [2]. Several proteomics studies of denervated skeletal muscle have also revealed biomarkers and pathways involved in denervation-induced muscle atrophy [3]. In spite of previous understanding of the mechanism for denervated nerves, the re-innervation response to crushed nerves has not been well characterized.

The effects of axotomy on motoneurons, nerves and muscles implicate various morphological, biochemical and physiological changes. After a nerve is transected, the damage axons will regenerate following their unique pathway, and eventually re-innervate the muscles. As previously described, nerve transection will redirect regenerated axons to inappropriate pathways [4]. In contrast to axotomy, the experimental technique of crushing maintains the stability of nerve sheaths and basement membranes. Despite the fact that advances have been made in clarifying the mechanisms of axon and muscle regeneration, the underlying mechanism for re-innervation has yet to be evaluated using a proteomic or transcriptomic approach.

Therefore, the current study aims to investigate possible functional mechanisms by identifying differentially expressed mRNA and proteins in re-innervated muscle over a 4-week period following injury. Furthermore, possible treatment targets might be identified by using bioinformatic analysis, including Gene Ontology (GO) terms and Kyoto Encyclopedia of Genes and Genomes (KEGG) pathway analysis. The 4-week study period covers the time when neurogenic muscular atrophy is still reversible and nerve re-innervation and myogenic regeneration can still occur [5, 6]. This research brings new insight into molecular mechanism of nerve re-innervation, while identifying specific targets and pathways for the novel treatment of nerve re-innervation.

## Results

### General observation

Daily observations of animals in each experimental group revealed normal behavior and no systemic or local inflammation or post-operative complications.

### Tibial function index (TFI) analysis

**Fig. 1:**
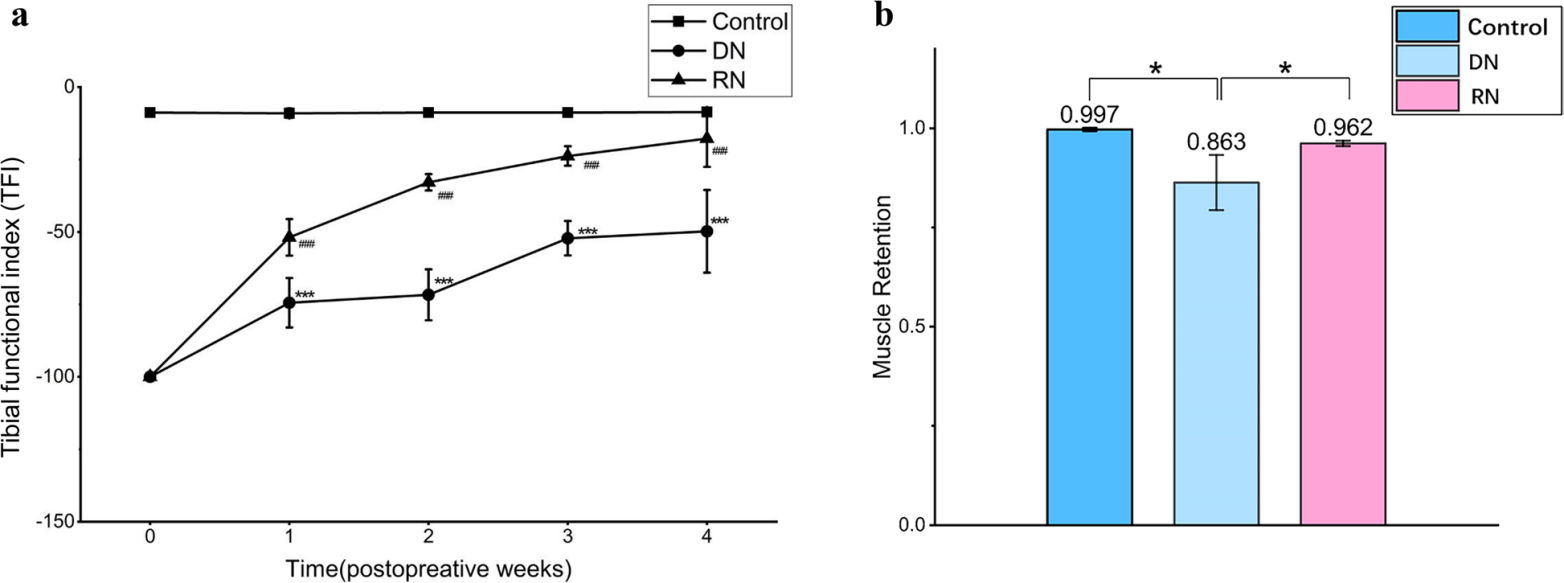
RN group achieved a higher TFI value and better protection for the gastrocnemius muscle. **a** The tibial functional index (TFI) was determined after analysis of the walking trajectory. The higher TFI values represent better functional recovery. #RN group versus control group, *DN group versus control group. Results statistically significant at *P* < 0.05 (*, #), *P* < 0.01 (**, ##) and *P* < 0.001 (***, ###). **b** The ratio of muscle on the surgical side compared to the healthy side. * Differences were statistically significant at *P* < 0.05. By 4 weeks, RN group revealed a well recovery of muscle compared with the DN (*P* < 0.01)

TFI analysis revealed that the TFI values decreased after denervation and crush, and repair increasingly with time. The TFI values in the RN and DN group were significantly lower than in the control group at every time point, respectively (*P* < 0.001, Fig. 1a). The TFI values in the RN group were significantly higher than in the DN group at every time point (*P* < 0.001, Fig. 1a).

### Muscle wet weight

The gastrocnemius muscle wet weight was scaled at 4 weeks (Fig. 1b). To deeply know the muscle mass, we compared the ratio of muscle samples between surgical and healthy side. The muscle mass of the control group is 100% retained. By 4 weeks, DN group revealed a poor return of muscle recording only 86.3% ± 7% (*P* < 0.05). In RN group, it was 96.2% ± 0.6% which was well recovered compared with the DN (*P* < 0.05).

### Differentially expressed genes (DEGs) and proteins (DEPs)

**Fig. 2:**
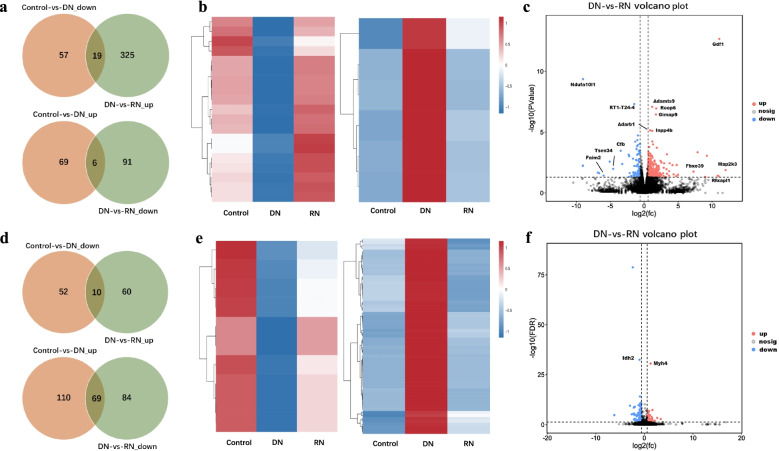
Signatures of DN and RN group at transcript and protein levels. **a** Venn-diagrams showing the number of differentially expressed genes (DEGs) that were down-regulated in the DN group (compared with the control group) and up-regulated in the RN group (compared with the DN group), and DEGs that were up-regulated in DN rats and down-regulated in RN rats. **b** Heatmap representation of hierarchical clustering of DEGs that were significantly altered in all three groups. **c** Volcano plot revealing the distribution of DEGs in the RN group compared to the DN group. **d** Venn-diagram revealing the number of differentially expressed proteins (DEPs) in DN group compared to control group, and DEPs in DN group compared to RN group. **e** Heatmap representation of hierarchical clustering of DEPs showing the proteins altered in all three groups. **f** Volcano plot showing distribution of the DEPs in RN group compared to the DN group

Untargeted transcriptome and proteome analyses of muscles were performed to explore repair mechanisms in the DN and RN groups (Fig. 2a). A total of 567 genes were identified as differentially expressed genes (DEGs) at the transcriptome level, out of which 151 were altered in DN mice relative to control mice, while 441 DEGs were altered through re-innervating (Fig. 2a). Upon comparing the DN group with the RN group, 344 DEGs were found to be significantly up-regulated, while 97 DEGs were significantly down-regulated. Compared with the control group, 19 genes were down-regulated in DN group, while restored through re-innervating. In the same way, 6 genes were up-regulated in DN group and restored through re-innervating. These results were visualized in the hierarchically clustered heatmap (Fig. 2b) and volcano plot (Fig. 2c). Heatmap representation of hierarchical clustering of DEGs that were significantly altered by RN.

At the proteome level, a total of 385 proteins were differentially expressed. The Venn diagram in Fig. 2d shows the number of differentially expressed proteins (DEPs) in all 3 groups. Among the 223 proteins that were significantly affected by RN, 70 were up-regulated and 153 were down-regulated. Compared with control group, 10 proteins were down-regulated in DN group, and then restored through re-innervating, while 69 proteins were up-regulated and restored through re-innervating. The corresponding heatmap and volcano plot are shown in Fig. 2e, f.

### Gene Ontology (GO) and Kyoto Encyclopedia of Genes and Genomes (KEGG) Pathways for the Differentially expressed genes (DEGs) and proteins (DEPs)

**Fig. 3:**
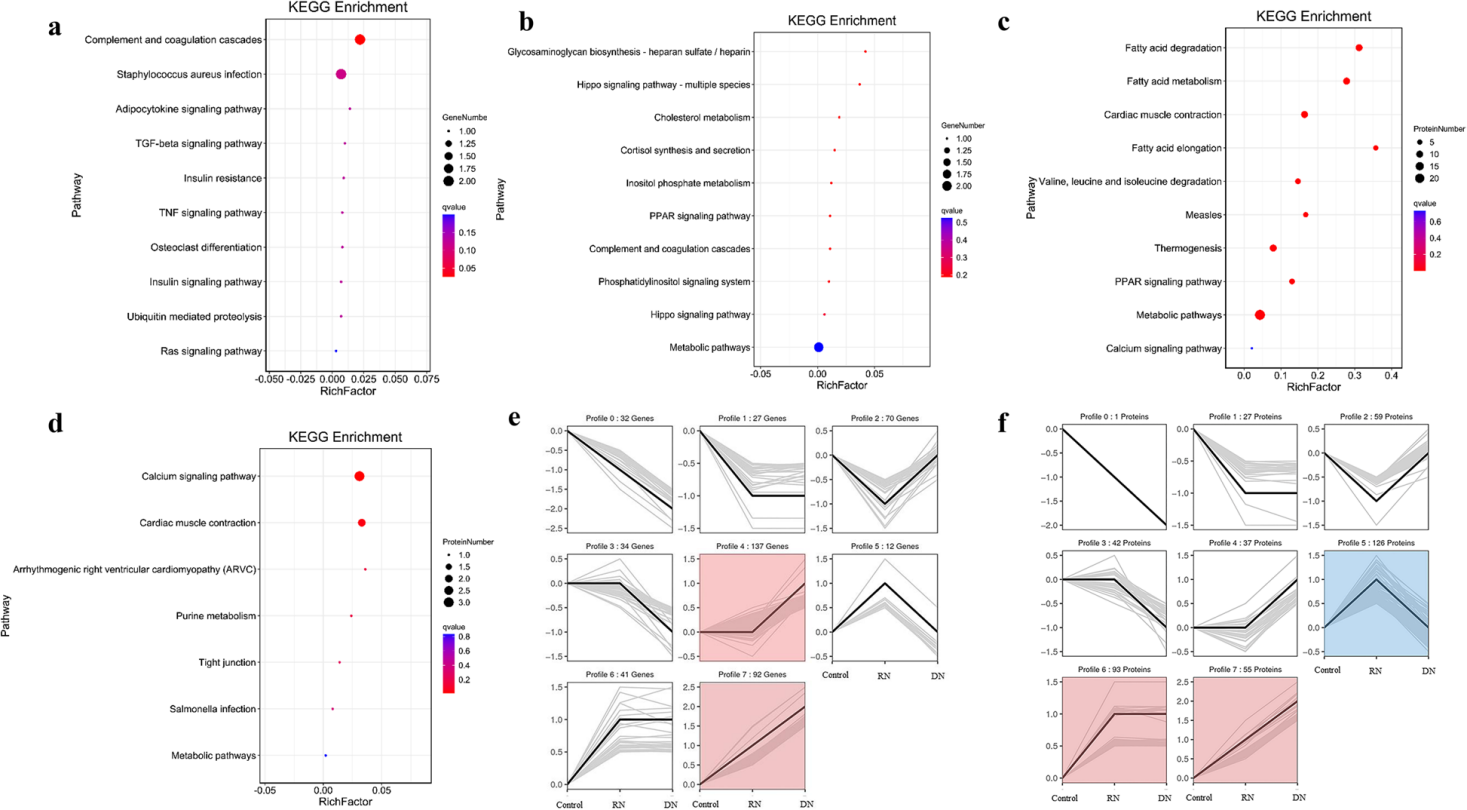
Network analysis of transcriptome and proteome. **a, b, c, d** Analysis of KEGG pathway enrichment in DEGs (**a**, **b**) and DEPs (**c**, **d**). Rich factor refers to the ratio of the number of DEGs (or DEPs) in the pathway entry to the total number of genes (or proteins) in that pathway entry. Pathways were considered significantly enriched when *P* < 0.05. **e, f** Short time-series expression miner (STEM) clustering of DEGs (Fig. **e**) and DEPs (Fig. **f**). Each square represents the expression trend of a gene (or protein) in the control, DN and RN groups. The black polygonal line in the square indicates the overall expression profile of all DEGs (or DEPs) in the corresponding set. The profile ID and the number of DEGs and DEPs in each profile are shown on the top of the square. Colored squares are significantly different (*P* < 0.05). DEPs involved in the energy metabolic pathway were mainly distributed in the expression patterns of profiles 5 in STEM analysis

Pathway enrichment was analyzed against KEGG database to obtain the potential functions of the DEGs and DEPs. As the results shown in Figs. 3a, b, c, d, DEGs and DEPs were all enriched in pathways involving in PPAR signaling pathway, while DEPs were significantly enriched in pathways related to energy metabolism. Among 126 DEPs distributed in the profile 5 of the short time-series expression miner (STEM) analysis (Fig. 3f), 55 DEPs were related to energy metabolism and suggested RN group a positive effect on energy metabolism comparing with DN group.

### Analysis combined transcriptome with proteome data

**Fig. 4:**
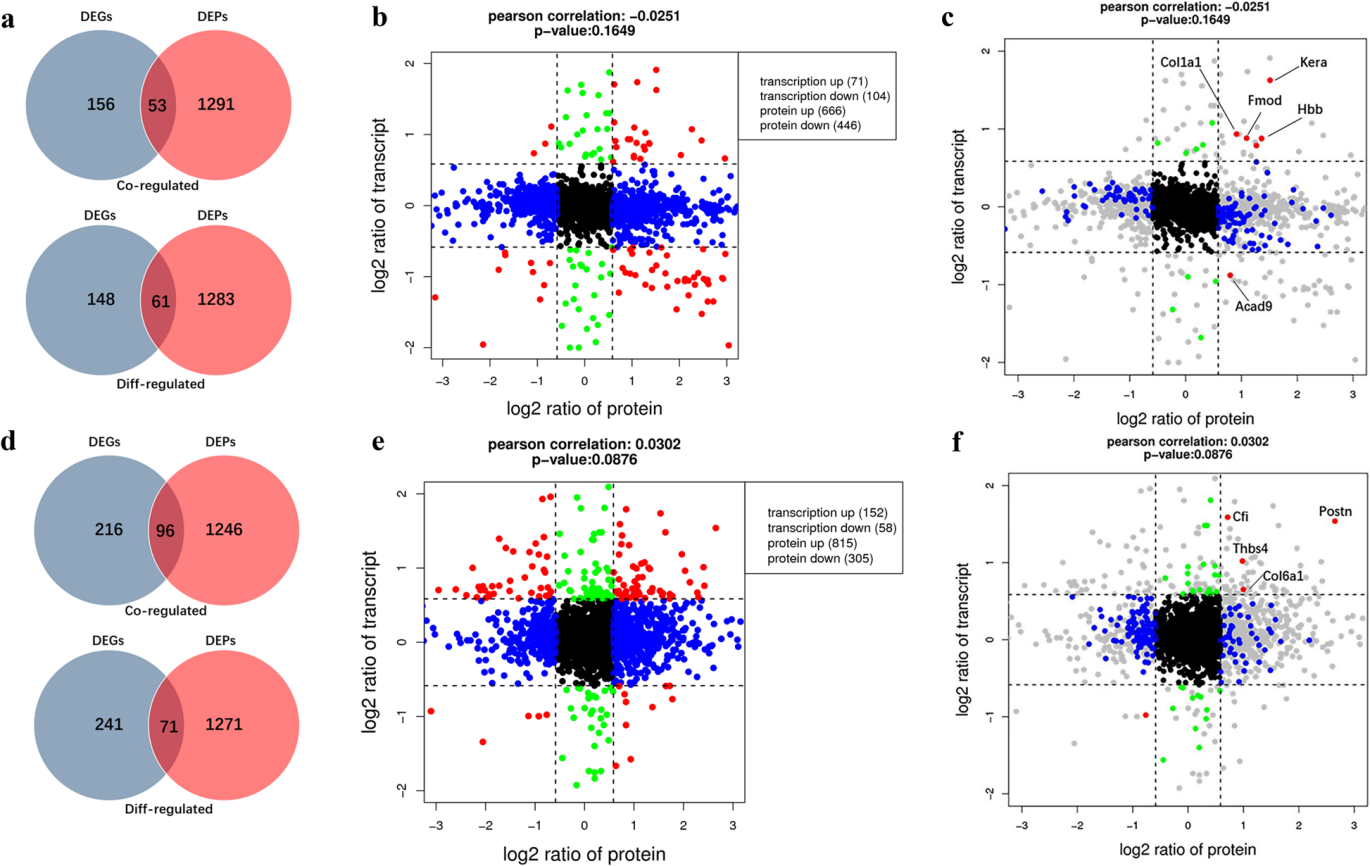
Correlational analysis of transcriptome and proteome. **a** Venn-diagrams showing the number of co-regulated DEGs/ DEPs, and diff-regulated DEGs/ DEPs between the DN group and control group. **b** DEGs and DEPs that were altered in both omics layers between the DN group and control group. **c** Correlational analysis of DEGs and DEPs in the DN group relative to the control group. The omics datasets were merged based on the gene symbol. Red dots in the Q3 or Q7 quadrants represent co-regulated entities that were consistently up-regulated or down-regulated. Green dots in the Q2 and Q8 quadrants represent genes that were significantly altered in the transcriptome, while blue dots in the Q4 and Q6 quadrants represent significant genes in the proteome (Fig. c, Q3). **d** Venn-diagram revealing the number of co-regulated DEGs/ DEPs, and diff-regulated DEGs/ DEPs between the DN group and RN group. **e** DEGs and DEPs that were altered in both omics layers between the RN group and DN group. **f** Correlational analysis of DEGs and DEPs in the RN group relative to the DN group

Furthermore, we combined the transcriptome and proteome data to identify genes that were altered in both omics’ layers. The Venn diagram in Fig. 4a shows the number of co-regulated DEGs/ DEPs, and diff-regulated DEGs/ DEPs between the DN group and control group. In the transcriptome level, 209 DEGs were up-or-down-regulated, while 1344 DEPs were up-or-down-regulated in the proteome level. Combined with transcriptome and proteome data, 53 were co-regulated while 61 were diff-regulated. Compared with RN group, 96 were co-regulated while 71 were diff-regulated (Fig. 4d). Of the 5 genes were found to be up-regulated in DN group (Fig. 4c, Q3), out of a total of 2534 genes co-regulated (Fig. 3b, Q3). In RN group, 4 genes differed significantly at both the protein and mRNA levels compared with DN group (Fig. 4f, Q3). These included *Myh3*, *Postn*, *Col6a1*, *Thbs4*, *Cfi* and so on, which are widely known to be related with muscle development, energy metabolism and neuronal differentiation.

## Discussion

Several studies support the notion that longer de-innervated periods cause reduced re-innervation and ultimately reduced the recovery of muscle function [7]. Significantly, the re-innervation of peripheral nerves is known to influence the recovery of denervated muscle [8]. These observations highlight the need to re-innervating muscles during the early recovery and before unrecoverable neural impairment occurs. Hence, there is considerable interest in identifying the molecular mechanisms of repair using nerve injury models so as to avoid irreversible outcomes post-operatively. Gene expression studies performed with various muscle injury models have reported that certain genes may be critical for achieving a good clinical result [9, 10]. To date, however, the importance of nerve integrity in the repair and recovery of nerve injury is not known. In the present work, the tibial nerve was crushed near the sciatic nerve branch, conducting a re-innervated model and studying gastrocnemius muscle specifically. Moreover, we conducted transcriptomic and proteomic analyses of muscle following nerve crush and transection in order to gain insights into the repair mechanisms following these injuries.

In the current study, transcriptomic and proteomic analysis of muscles were evaluated at 4 weeks after the nerve injury. The different degrees of nerve injury applied to the DN and RN groups appears to result in the different expression profiles of genes and proteins, as well as the muscle repair process. We proved that RN group achieved better functional results concerning the peripheral nerve injury model of rats than DN group. The tibial nerve function of the RN group recovered better with a higher TFI value and better protection for the gastrocnemius muscle. Transection of nerve caused a greater degree of axonal destruction, which required greater recovery and re-innervation at the lesion site. In contrast with transection, nerve crush preserved the nerve sheath conduit, so as to allow the axonal regeneration and ultimately evoke a different genes profile. Apart from the Venn-diagrams, volcano plot in Fig. 2c was also demonstrated the different gene profiles between DN and RN groups at 4 weeks post-repair. Furthermore, pathway enrichment analysis based on the KEGG database was performed to evaluate the potential function of DEGs and DEPs. As shown in Fig. 3c, DEPs were significantly enriched in pathways related to energy metabolism, such as fatty acid metabolism, degradation and elongation. Interestingly, the DEPs involved in energy metabolic pathway were mainly distributed in the expression patterns of profiles 5 in STEM analysis (Fig. 3f), which suggest that RN group had a positive effect on energy metabolism comparing to DN group.

When the peripheral nerve is damaged, the distal axons of the injured site degenerates, then Schwann cells of peripheral nerve are able to proliferate and re-differentiate, thus providing an environment for axonal and nerve regeneration [11]. As Zhang et al. reported, Peroxisome proliferator-activated receptors-γ (PPAR-γ) expression in SCs increases during the inflammation process and this may promote their activation as immune cells [12]. In the current study, the PPAR pathway was found to be one of the major signaling pathways according to the multi-omics analyses. They regulate genes that are critical for cellular differentiation, lipid and metabolic homeostasis, and energy balance [13, 14]. PPAR-γ plays a key role in muscle cell function and myogenesis, which expressed in the skeletal muscle of humans [15]. As a nuclear receptor, it can activate cellular metabolism to promote cell growth and differentiation [16, 17]. Given these observations, drugs that activate PPAR-γ signaling pathway may therefore be effective in reducing and preventing muscle atrophy.

Cellular respiration is a series of metabolic reactions that convert biochemical energy to ATP and then release the waste products [18, 19]. Skeletal muscle cells efficiently converted energy through several metabolic pathways to maintain continuous muscle contraction. In denervated muscle, the process by which mitochondria produce ATP is damaged. Consistent with earlier reports [20], we found lower expression levels for almost all metabolic pathways after nerve denervation. In pathological conditions, the ability to regulate energy metabolism of skeletal muscle is disrupted. This may explain why many pathways associated with energy metabolism were found to be enriched in the current research. Furthermore, the levels of ATP increase rapamycin (mTOR) activity, and the mammalian target of mTOR pathway is the latest intracellular fuel-sensing mechanism to be implicated in the regulation of energy balance [21–23]. In organism, mTOR was a key regulator in maintaining skeletal muscle mass which regulates protein anabolism and catabolism [24]. We have previously proven that negative regulation of mTORC1 signaling could protect the denervated muscle atrophy and improved the functional outcome [25–28]. The essence of denervated muscle atrophy results from more significant muscle protein degradation than synthesis [29, 30]. After denervation, mTORC1 signaling plays more important roles in protein degradation than in protein synthesis and leads to muscle atrophy [24]. Recent studies have also demonstrated that mTORC1 activation increases both oxygen consumption and mitochondrial number [31], it might have a significant role in the coordination of cell growth and energy metabolism. Combined with previous studies, the changes in genes and proteins involved in energy metabolism we investigated in the present study, may be associated with the mTOR pathway, leading to muscle atrophy and thus reduced muscle function.

In the rat model used here, a significant up-regulation of thrombospondin 4 (*Thbs4*) in RN muscles was observed in the 4-week post-surgery period, which has important functions for neurogenesis in early post-natal and adult rodent brain [32]. *Thbs4* could enhance the vesicular trafficking of membrane attachment complexes to stabilize the sarcolemma in myofibers [33]. On the other hand, Thbs4 homozygous knockout mice (Thbs4-KO) resulted in abnormal glial scar formation after injury, and significantly increased microvascular haemorrhage into the brain parenchyma [32]. Loss of *Thbs4* expression led to dystrophic changes in the muscular dystrophic mouse models, while overexpression protected muscle and alleviated dystrophic disease.

Other nerve growth factors with increased expression observed in the RN group included Myh3, as well as Postn, Col6a1, and Cfi. Myosin heavy chain 3 (*Myh3*) gene is an important component of myosin, which is related to the traction and sliding of muscle [34]. It is highly induced during in vitro muscle cell differentiation, and its expression is markedly increased in muscular dystrophy, where it is thought to represent a marker of muscle regeneration [35]. As an important marker gene for muscle regeneration, *Myh3* was also found high expression in the RN group in our study, and its expression correlated with the time when most muscle repair occurs [36]. In addition, *Myh3* had also been reported to express during fetal development and muscle regeneration [37]. Periostin (*Postn*) is an extracellular matrix protein secreted by osteoblast and osteoblast-like cell line, which can promote proliferation and differentiation of osteoblast and its precursors on the periosteum [38]. It can not only promote the reconstruction of damaged blood vessels [39], but also promote the hyperplasia and fibrosis of inflammatory tissue [40]. Basing on above evidences, this may be related to the process of muscle repair and nerve regeneration in the RN group.

Collagen type VI alpha 1 chain (*Col6α1*) is widely distributed in the extracellular matrix of systemic tissues including skeletal muscle. Deficiency of *Col6α1* has been shown to cause mitochondrial defects (25–30) and impaired muscle regeneration [41]. As Sakurai et al. showed [42], *Col6α1* supplementation promoted muscle regeneration and maturation in *Col6a1*-KO mice. Thus, the high expression of *Col6α1* in RN group might relate to the better muscle recovery in the present study. As for the complement factor I (*Cfi*), local complement expression by skeletal muscle in vivo may be implicated in some muscular inflammatory or pathological processes. As shown in the above studies, these genes are involved in regulating the muscle development or regeneration in vitro, however, the specific mechanism needs to be further studied and allow them to be the possible test candidates for therapeutic application.

It should be mentioned that there are some limitations in this study. The rats used in the experiments were female at about 8 weeks, which are between the puberty stage and breeding age. The phase of estrous cycle may have an influence on the secretion of various hormones that regulate muscle metabolism. After crushing nerve injury, temporary activity limitations may also affect food intake, which may in turn affect gene and muscle protein expression. However, the rats used in each group are the same age, which can ensure the consistency of each group and minimize the influence of hormonal profile between each group. Another drawback of our study was genes and related pathways were not investigated. The detection of candidate genes and proteins are not enough to support the whole survey for obtaining more comprehensive information. Perhaps, hormone secretion levels of rats and functional analyses of key genes/ proteins could be included in the future study to obtain more comprehensive information.

In spite of the mechanisms of muscle atrophy are still not fully understood, new approaches are now available to comprehensively observe changes in gene and protein expression during skeletal muscle atrophy caused by crush or transection. This has prompted investigations into novel therapeutic and biological solutions to enhance neural repair at an earlier phase. We identified 441 DEGs and 223 DEPs that were specific to the way of nerve injury. Furthermore, several possible candidate genes were identified in the re-innervated nerve, including *Thbs4*, *Myh3*, *Postn*, *Col6a1* and *Cfi*. In particular, the PPAR signaling and energy metabolism pathways seems to play a vital role and warrant further study. This study provides basic information on the changes that occur in gene and protein expression during re-innervation-induced muscle atrophy and identified several key proteins as possible treatment targets. The results form the basis for further research aimed at the prevention and therapy of muscle atrophy.

## Methods and materials

### Animals

Eighteen adult female Sprague–Dawley rats (eight weeks, 200–250 g) were provided by the Experimental Animal Center of South China University of Technology and randomly divided into 3 groups (*n* = 6) to undergo nerve operations or sham operations. All experiments were carried out in SPF (specific pathogen free) animal house, and the animal feed was formulated based on the nutritional requirements of rats issued by the Ministry of Health.

### Surgical procedures

We randomly divided the rats into three groups: the denervated (DN) group, the re-innervated (RN) group, and the control group. Standard aseptic technique was conducting to perform the surgery on the right posterior limb of each animal. Briefly, adult rats were anesthetized with the 30 mg/kg dose of sodium pentobarbital by intraperitoneal injection. All procedures were performed on animals in an unconscious stage on the same day. A longitudinal incision was made posterior to expose the sciatic nerve, and free the tibial and peroneal nerves totally. For relieving post-surgery pain, the rats were given buprenorphine (0.2 mg/kg subcutaneously) when performing the operation.

**Fig. 5:**
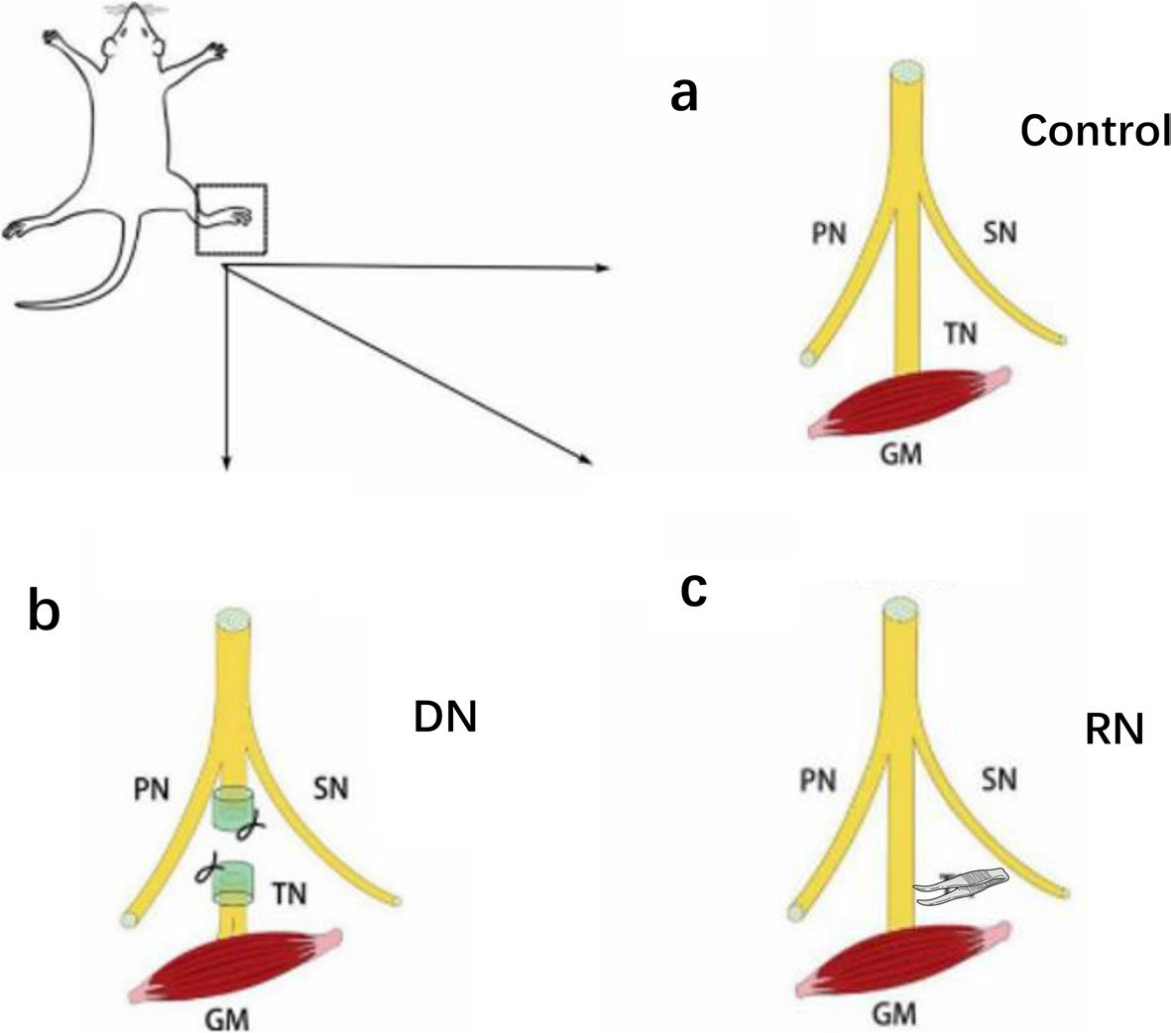
Experimental nerve injury models. The two simulated nerve injuries were the DN group for denervated nerve (nerve was transected), and the RN group for re-innervated nerve (nerve was crushed to damage the axons). In the control group, the nerve was preserved intact. All muscle lacerations were repaired with sutures

In the control group, the tibial nerve of rats was exposed clearly without being cut (Fig. 5a). In the DN group, the tibial nerve was transected at 6.5 mm proximal to the gastrocnemius muscle, thus simulating a denervated muscle. In order to prevent spontaneous re-innervation of the tibial nerve, we covered the proximal and distal nerve stumps with a silicone cap (Fig. 5b). In the RN group, the exposed tibial nerve at the same location as the transection site in the DN group was crushed three times for 10 s each using a pair of forceps, with an interval of 10 s. This model simulates a re-innervated muscle (Fig. 5c). The surgical wounds of all rats were thoroughly irrigated and sutured in layers. After the surgical incisions closed, animals were housed in controlled, maintaining a 12-h light/12-h dark cycles in the pathogen-free condition and allowed free access to water and food.

### Analysis of Tibial Function Index (TFI)

TFI were calculated before and every week after surgery. After dipping the hindlimbs into black ink, the rats walked on the confined walking track (10 cm × 60 cm) covering with white paper. After the test, the footprint on the track was measured with the standard of clarity and completeness. We recorded the paired footprint length (PL, distance from heel to toe), intermediary toe spread (IT, distance from second to fourth toes), and toe spread (TS, distance from first to fifth toes) of each animal’s hind limb. The lengths of normal control left hind leg were defined as NPL, NIT, and NTS, while the experimental right legs were EPL, EIT, and ETS. The TFI analysis was performed following the formula of Bain-Mackinnon-Hunter [43]:

$$\mathrm{TFI }=104.4 \left(\left[\mathrm{ETS}-\mathrm{NTS}\right]/\mathrm{NTS}\right)- 37.2 \left(\left[\mathrm{EPL}-\mathrm{NPL}\right]/\mathrm{NPL}\right)+ 45.6 \left(\left[\mathrm{EIT}-\mathrm{NIT}\right]/\mathrm{NIT}\right) -8.8$$

### Muscle weight

Four weeks after the surgery, the animals were sacrificed by the lethal dose of ether. Three operated muscle samples from each group were randomly selected to peel the gastrocnemius muscle and tibial nerve completely. The muscle wet weight was scaled analytical balance (BSA124S, sartorius). The muscle samples were snap-frozen in liquid nitrogen for more than 15 min, and stored at -80℃ until bioinformatic analysis 3 days later.

### Transcriptomic analysis

#### RNA extraction, library construction and sequencing

Trizol reagent kit (Invitrogen, Carlsbad, CA, USA) was used to extract all RNA. Oligo(dT) beads were used to enrich eukaryotic mRNA, while Ribo-ZeroTM Magnetic Kit (Epicentre, Madison, WI, USA) was used to remove rRNA and enrich prokaryotic mRNA. The enriched mRNA was reverse transcribed into cDNA using primers. DNA polymerase I, RNase H, dNTP and buffer were used to synthesize second-strand cDNA, and purified using PCR extraction kit (Qiagen, Venlo, The Netherlands). The sequencing platform of Genedenovo Biotechnology Co., Ltd (Guangzhou, China) is responsible for constructing the library and sequencing it.

#### Gene Ontology (GO) and Kyoto Encyclopedia of Genes and Genomes (KEGG) pathways enrichment analysis

Differential expression analysis of mRNA between two groups was performed using the R package DESeq2, and between two samples by edgeR. Statistically significant differentially expressed genes were detected if parameter of false discovery rate (FDR) set at < 0.05 and an absolute fold change ≥ 2. The KEGG analysis was performed using the KEGG pathway database (Release 94, https://www.genome.jp/kegg/pathway.html) to analyze the selected DEGs at the functional level [44–46].

### Proteomic analysis

#### Protein digestion

The gastrocnemius muscle samples were put into the lysis buffer (1 mg/ml protease inhibitor, 7 M urea, 2% SDS), The protein concentration of the supernatant was measured using BCA Protein Assay Kit. 50 μg proteins were suspended in 50μL solution, 1uL 1 M dithiotreitol was added for reduction at 55 °C for 1 h, and 5uL 20 mM iodoacetamide was added for alkylation at 37 °C for 1 h. Then 300uL acetone was chilled and precipitated the sample overnight at -20℃. After two washes with cold acetone, the precipitate was resuspended in 50 mM ammonium bicarbonate. Finally, sequence-grade modified trypsin (Promega, Madison, WI) was used to digest the proteins at a substrate/enzyme ratio of 50:1 (w/w) at 37 °C for 16 h.

#### High PH Reverse Phase Separation

By using high pH separation, the peptide mixture was fractionated on an Ultimate 3000 UPLC system (Thermo Fisher Scientific, Waltham, MA, USA), then analyzed on EASY-nLC 1200 system (Thermo Fisher Scientific, MA, USA).

#### Data analysis

Spectronaut X (Biognosys AG, Switzerland) analyzed the raw Data of DIA with default parameters. DEPs with Absolute AVG log2 ratio > 0.58 and *q* < 0.05 will be filtered after Student’s T-test. KEGG database were annotated to obtain function of proteins, while GO pathways were detected within DEPs with *q* < 0.05.

### Statistical analysis

Continuous data were presented as mean ± standard deviation or median with interquartile range. Two-tailed tests were used, and differences were considered significant if *p* < 0.05. For experiments on more than two groups within one categorical variable, ANOVA analysis was performed followed by Tukey´s post hoc test. SPSS 20.0 statistical software (Chicago, IL, USA) was used for the analysis. The data that support the findings of this study are available from the corresponding author upon reasonable request.

## Data Availability

The mass spectrometry proteomics data have been deposited to the ProteomeXchange Consortium (http://proteomecentral.proteomexchange.org) via the iProX partner repository [47] with the dataset identifier PXD033031.

## Abbreviations

DN: Denervated
RN: Re-innervated
RNA seq: RNA sequencing
SCs: Schwann cells
PPARs: Peroxisome proliferator-activated receptors
DEGs: Differentially expressed genes
DEPs: Differentially expressed proteins
GO: Gene Ontology
KEGG: Kyoto Encyclopedia of Genes and Genomes
*Myh3*: Myosin Heavy Chain 3
*Postn*: Periostin gene
*Col6a1*: Collagen type VI alpha 1 chain
*Cfi*: Complement factor I
mTOR: Rapamycin

## References

[1] Russo TL, Peviani SM, Durigan JL, Gigo-Benato D, Delfino GB, Salvini TF (2010). Stretching and electrical stimulation reduce the accumulation of MyoD, myostatin and atrogin-1 in denervated rat skeletal muscle. J Muscle Res Cell Motil.

[2] Weng J, Zhang P, Yin X, Jiang B (2018). The whole transcriptome involved in denervated muscle atrophy following peripheral nerve injury. Front Mol Neurosci.

[3] Lang F, Aravamudhan S, Nolte H, Türk C, Hölper S, Müller S, Günther S, Blaauw B, Braun T, Krüger M (2017). Dynamic changes in the mouse skeletal muscle proteome during denervation-induced atrophy. Dis Model Mech.

[4] Zhou CJ, Kawabuchi M, Wang S, Liu WT, Hirata K (2002). Age differences in morphological patterns of axonal sprouting and multiple innervation of neuromuscular junctions during muscle reinnervation following nerve crush injury. Ann Anat.

[5] Pereira BP, Han HC, Yu Z, Tan BL, Ling Z, Thambyah A, Nathan SS (2010). Myosin heavy chain isoform profiles remain altered at 7 months if the lacerated medial gastrocnemius is poorly reinnervated: a study in rabbits. J Orthop Res.

[6] Shen Y, Zhang R, Xu L, Wan Q, Zhu J, Gu J, Huang Z, Ma W, Shen M, Ding F (2019). Microarray analysis of gene expression provides new insights into denervation-induced skeletal muscle atrophy. Front Physiol.

[7] Aydin MA, Mackinnon SE, Gu XM, Kobayashi J, Kuzon WM (2004). Force deficits in skeletal muscle after delayed reinnervation. Plast Reconstr Surg.

[8] Carlson BM (2008). The denervated muscle: 45 years later. Neurol Res.

[9] Zeman RJ, Zhao J, Zhang Y, Zhao W, Wen X, Wu Y, Pan J, Bauman WA, Cardozo C (2009). Differential skeletal muscle gene expression after upper or lower motor neuron transection. Pflugers Arch.

[10] Warren GL, Summan M, Gao X, Chapman R, Hulderman T, Simeonova PP (2007). Mechanisms of skeletal muscle injury and repair revealed by gene expression studies in mouse models. J Physiol.

[11] Fu SY, Gordon T (1997). The cellular and molecular basis of peripheral nerve regeneration. Mol Neurobiol.

[12] Zhang F, Liu F, Yan M, Ji H, Hu L, Li X, Qian J, He X, Zhang L, Shen A (2010). Peroxisome proliferator-activated receptor-gamma agonists suppress iNOS expression induced by LPS in rat primary Schwann cells. J Neuroimmunol.

[13] Corton JC, Anderson SP, Stauber A (2000). Central role of peroxisome proliferator-activated receptors in the actions of peroxisome proliferators. Annu Rev Pharmacol Toxicol.

[14] Kersten S, Desvergne B, Wahli W (2000). Roles of PPARs in health and disease. Nature.

[15] Kruszynska YT, Mukherjee R, Jow L, Dana S, Paterniti JR, Olefsky JM (1998). Skeletal muscle peroxisome proliferator- activated receptor-gamma expression in obesity and non- insulin-dependent diabetes mellitus. J Clin Invest.

[16] Hihi AK, Michalik L, Wahli W (2002). PPARs: transcriptional effectors of fatty acids and their derivatives. Cell Mol Life Sci.

[17] Murphy GJ, Holder JC (2000). PPAR-gamma agonists: therapeutic role in diabetes, inflammation and cancer. Trends Pharmacol Sci.

[18] O'Leary MF, Hood DA (2008). Effect of prior chronic contractile activity on mitochondrial function and apoptotic protein expression in denervated muscle. J Appl Physiol (1985).

[19] Batt J, Bain J, Goncalves J, Michalski B, Plant P, Fahnestock M, Woodgett J (2006). Differential gene expression profiling of short and long term denervated muscle. FASEB J.

[20] Adhihetty PJ, O'Leary MF, Chabi B, Wicks KL, Hood DA (2007). Effect of denervation on mitochondrially mediated apoptosis in skeletal muscle. J Appl Physiol (1985).

[21] Dennis PB, Jaeschke A, Saitoh M, Fowler B, Kozma SC, Thomas G (2001). Mammalian TOR: a homeostatic ATP sensor. Science.

[22] Cota D, Proulx K, Smith KA, Kozma SC, Thomas G, Woods SC, Seeley RJ (2006). Hypothalamic mTOR signaling regulates food intake. Science.

[23] Ropelle ER, Pauli JR, Fernandes MF, Rocco SA, Marin RM, Morari J, Souza KK, Dias MM, Gomes-Marcondes MC, Gontijo JA (2008). A central role for neuronal AMP-activated protein kinase (AMPK) and mammalian target of rapamycin (mT OR) in high-protein diet-induced weight loss. Diabetes.

[24] Yoon MS (2017). mTOR as a Key Regulator in Maintaining Skeletal Muscle Mass. Front Physiol.

[25] Chen Y, Yuan W, Zeng X, Ma Y, Zheng Q, Lin B, Li Q (2021). Combining reverse end-to-side neurorrhaphy with rapamycin treatment on chronically denervated muscle in rats. J Integr Neurosci.

[26] Li QT, Zhang PX, Yin XF, Han N, Kou YH, Deng JX, Jiang BG (2013). Functional recovery of denervated skeletal muscle with sensory or mixed nerve protection: a pilot stu dy. PLoS One.

[27] Li Q, Zhang P, Yin X, Han N, Kou Y, Jiang B (2014). Early sensory protection in reverse end-to-side neurorrhaphy to improve the functional recovery of ch ronically denervated muscle in rat: a pilot study. J Neurosurg.

[28] Li Q, Zhang P, Yin X, Jiang B (2015). Early nerve protection with anterior interosseous nerve in modified end-to-side neurorrhaphy repairs high ulnar nerve injury: a hypothesis of a novel surgical technique. Artif Cells Nanomed Biotechnol.

[29] Kamei Y, Miura S, Suzuki M, Kai Y, Mizukami J, Taniguchi T, Mochida K, Hata T, Matsuda J, Aburatani H (2004). Skeletal muscle FOXO1 (FKHR) transgenic mice have less skeletal muscle mass, down-regulated Type I (s low twitch/red muscle) fiber genes, and impaired glycemic control. J Biol Chem.

[30] Bonaldo P, Sandri M (2013). Cellular and molecular mechanisms of muscle atrophy. Dis Model Mech.

[31] Finley LW, Haigis MC (2009). The coordination of nuclear and mitochondrial communication during aging and calorie restriction. Ageing Res Rev.

[32] Benner EJ, Luciano D, Jo R, Abdi K, Paez-Gonzalez P, Sheng H, Warner DS, Liu C, Eroglu C, Kuo CT (2013). Protective astrogenesis from the SVZ niche after injury is controlled by Notch modulator Thbs4. Nature.

[33] Vanhoutte D, Schips TG, Kwong JQ, Davis J, Tjondrokoesoemo A, Brody MJ, Sargent MA, Kanisicak O, Yi H, Gao QQ (2016). Thrombospondin expression in myofibers stabilizes muscle membranes. eLife.

[34] Knight AE, Molloy JE (2000). Muscle, myosin and single molecules. Essays Biochem.

[35] Haslett JN, Sanoudou D, Kho AT, Bennett RR, Greenberg SA, Kohane IS, Beggs AH, Kunkel LM (2002). Gene expression comparison of biopsies from Duchenne muscular dystrophy (DMD) and normal skeletal muscle. Proc Natl Acad Sci USA.

[36] Chargé SB, Rudnicki MA (2004). Cellular and molecular regulation of muscle regeneration. Physiol Rev.

[37] Karsch-Mizrachi I, Travis M, Blau H, Leinwand LA (1989). Expression and DNA sequence analysis of a human embryonic skeletal muscle myosin heavy chain gene. Nucleic Acids Res.

[38] Litvin J, Selim AH, Montgomery MO, Lehmann K, Rico MC, Devlin H, Bednarik DP, Safadi FF (2004). Expression and function of periostin-isoforms in bone. J Cell Biochem.

[39] Li P, Oparil S, Feng W, Chen YF (2004). Hypoxia-responsive growth factors upregulate periostin and osteopontin expression via distinct signaling pathways in rat pulmonary arterial smooth muscle cells. J Appl Physiol (1985).

[40] Jia G, Erickson RW, Choy DF, Mosesova S, Wu LC, Solberg OD, Shikotra A, Carter R, Audusseau S, Hamid Q (2012). Periostin is a systemic biomarker of eosinophilic airway inflammation in asthmatic patients. J Allergy Clin Immunol.

[41] Alexeev V, Arita M, Donahue A, Bonaldo P, Chu ML, Igoucheva O (2014). Human adipose-derived stem cell transplantation as a potential therapy for collagen VI-related congenital muscular dystrophy. Stem Cell Res Ther.

[42] Takenaka-Ninagawa N, Kim J, Zhao M, Sato M, Jonouchi T, Goto M, Yoshioka CKB, Ikeda R, Harada A, Sato T (2021). Collagen-VI supplementation by cell transplantation improves muscle regeneration in Ullrich congenital muscular dystrophy model mice. Stem Cell Res Ther.

[43] Bain JR, Mackinnon SE, Hunter DA (1989). Functional evaluation of complete sciatic, peroneal, and posterior tibial nerve lesions in the rat. Plast Reconstr Surg.

[44] Kanehisa M, Goto S (2000). KEGG: kyoto encyclopedia of genes and genomes. Nucleic Acids Res.

[45] Kanehisa M (2019). Toward understanding the origin and evolution of cellular organisms. Protein Sci.

[46] Kanehisa M, Furumichi M, Sato Y, Ishiguro-Watanabe M, Tanabe M (2021). KEGG: integrating viruses and cellular organisms. Nucleic Acids Res.

[47] Ma J, Chen T, Wu S, Yang C, Bai M, Shu K, Li K, Zhang G, Jin Z, He F (2019). iProX: an integrated proteome resource. Nucleic Acids Res.

